# Inhibitory Effect of Polyphenol-Rich Fraction from the Bark of *Acacia mearnsii* on Itching Associated with Allergic Dermatitis

**DOI:** 10.1155/2012/120389

**Published:** 2012-01-22

**Authors:** Nobutomo Ikarashi, Wataru Sato, Takahiro Toda, Makoto Ishii, Wataru Ochiai, Kiyoshi Sugiyama

**Affiliations:** Department of Clinical Pharmacokinetics, Hoshi University, 2-4-41 Ebara, Shinagawa-ku, Tokyo 142-8501, Japan

## Abstract

We examined the inhibitory effect of polyphenol-rich aqueous extract from the bark of *Acacia mearnsii* (PrA) on itching associated with atopic dermatitis (AD). HR-1 mice were fed a normal diet, special diet (AD group), or special diet containing 3% PrA (PrA group) for 6 weeks. In the AD group, itching frequency and transepidermal water loss increased compared to the control group. In the PrA group, an improvement in atopic dermatitis symptoms was observed. Ceramide expression in the skin decreased in the AD group compared to the control group, but no decrease was observed in the PrA group. mRNA expression of ceramidase decreased in the PrA group compared to the AD group. The results of this study have revealed that PrA inhibits itching in atopic dermatitis by preventing the skin from drying. It is considered that the mechanism by which PrA prevents the skin from drying involves the inhibition of increased ceramidase expression associated with atopic dermatitis.

## 1. Introduction

Acacia is an evergreen tree belonging to the genus *Acacia* in the legume family, and it is widely distributed throughout the Australian and African continents. The extract of *Acacia catechu* duramen is called gambir, and it has long been used as an astringent and antibacterial to treat stomatitis and diarrhoea in Japan and China. In Europe, polyphenol-rich aqueous extract from the bark of *Acacia mearnsii* De Wild. (PrA), which is widely distributed throughout South Africa, has been used to eliminate wine sediment. Australian aborigines also eat the young leaves and beans of *Acacia mearnsii* De Wild. 

We have already demonstrated that PrA exerts antiobesity and antidiabetic actions by altering the expression of genes associated with the inhibition of obesity and diabetes in skeletal muscle, liver, and white adipose tissues [1]. We have also shown that PrA inhibits lipase and glucosidase activities, resulting in the inhibition of an increase in the plasma triglyceride and glucose levels [2].

In recent years, it has been reported that polyphenols exert various pharmacological actions, including antiobesity actions [3, 4], antidiabetic actions [5], and antiallergy actions [6, 7]. PrA is rich in unique catechin-like flavan-3-ols, such as robinetinidol and fisetinidol [8]. The catechins such as tea polyphenol [9, 10] and apple polyphenol [11] are effective in treating atopic dermatitis to some extent. Therefore, it is considered that PrA may also possibly exhibit antiatopic effects. To induce atopic dermatitis-like symptoms in this study, HR-1 hairless mice were given a special diet that does not contain magnesium [12, 13]. The inhibitory effect of PrA on itching associated with atopic dermatitis was examined using these mice.

## 2. Materials and Methods

### 2.1. Hot Water Extraction from Acacia Bark

PrA was donated by Mimozax Co., Ltd. (Hiroshima, Japan) and prepared according to the methods reported by Cutting [14]. Briefly, the powdered bark of South African *Acacia mearnsii* De Wild. was pulverised and extracted for 30 min in a 10-fold volume of hot water (100°C) and then dried using a spray drier. The extract yield was approximately 35% (w/w).

The polyphenol content of the present product as measured by the Stiasny reaction was 79.0%. The average molecular weight of PrA is 1,250 (300–3,000), and PrA contains robinetinidol, fisetinidol, syringic acid, gallocatechin, catechin, robinetinidol-(4*α*,8)-gallocatechin, and so forth [8]. PrA does not contain magnesium.

### 2.2. Materials

Propidium iodide (PI) solution was purchased from Wako Pure Chemicals (Osaka, Japan). Bovine serum albumin (BSA), mouse anticeramide antibody, and TRI reagent were purchased from Sigma-Aldrich Corp. (St. Louis, MO, USA). Rabbit anti-rat AQP3 antibody was purchased from Alomone Labs (Jerusalem, Israel). Alexa Fluor 488 anti-rabbit IgG, Alexa Fluor 488 anti-mouse IgM antibody, and primers were purchased from Invitrogen Corp. (Tokyo, Japan). Anti-rabbit IgG-HRP antibody was purchased from Santa Cruz Biotechnology Inc. (Santa Cruz, CA, USA). An enhanced chemiluminescence system (ECL) plus western blotting detection reagents were purchased from GE Healthcare (Chalfont St. Giles, UK). A high capacity cDNA synthesis kit was purchased from Applied Biosystems (Foster City, CA, USA). An iQ SYBR green supermix was purchased from Bio-Rad Laboratories (Hercules, CA, USA). All other reagents were of the highest commercially available grade.

### 2.3. Animals and Treatments

HR-1 hairless mice (4 weeks old) were purchased from Hoshino laboratory animals, Corp. (Ibaraki, Japan). Mice were kept at room temperature (24 ± 1°C) and 55 ± 5%  humidity with 12 h of light (artificial illumination: 08:00–20:00). The present study was conducted in accordance with the Guiding Principles for the Care and Use of Laboratory Animals, as adopted by the Committee on Animal Research at Hoshi University.

Mice that had been acclimated for 1 week were divided into four groups. Each group was provided with ad libitum access to a normal diet (Table 1, Nosan Corp., Kanagawa, Japan), commercial special diet (HR-AD diet, Table 1, Nosan Corp.), HR-AD diet containing 1.5% (w/w) PrA, or HR-AD diet containing 3.0% (w/w) PrA for 6 weeks. After 6 weeks, the skin was removed, frozen in liquid nitrogen, and stored at −80°C.

### 2.4. Analysis of Spontaneous Scratching Behaviour [12, 13]

At 4 and 6 weeks after the administration, we observed the itching behaviour in mice. The spontaneous itching behaviour of mice was videotaped for 1 h with a video camera. The scratching, grooming, and licking behaviour was observed by watching the videotape.

### 2.5. Measurement of Dermal Water Content and Transepidermal Water Loss

At 6 weeks after the administration, the dermal water content was measured using a Tewameter (TM300, Courage & Khazaka, Cologne, Germany). The transepidermal water loss was measured using a Corneometer (CM825, Courage & Khazaka). These measurements were carried out at 23 ± 1°C and 60 ± 10% humidity.

### 2.6. RNA Preparation from Tissue Samples

RNA was extracted from about 20 mg of frozen skin using the TRI reagents. RNA extraction was performed according to the protocol for the TRI reagents. The resulting solution was diluted 50-fold using Tris/EDTA buffer (TE buffer), and the purity and concentration (*μ*g/mL) of RNA were calculated by measuring absorbances at 260 and 280 nm using a U-2800 spectrophotometer (Hitachi High Technologies, Tokyo, Japan).

### 2.7. Real-Time RT-PCR

A high-capacity cDNA synthesis kit was used to synthesise cDNA from 1 *μ*g of RNA. TE buffer was used to dilute the cDNA 20-fold to prepare the cDNA TE buffer solution. The expression of target genes was detected by preparing the primers listed in Table 2 and by performing real-time RT-PCR. To each well of a 96-well PCR plate, 25 *μ*L of iQ SYBR green supermix, 3 *μ*L of forward primer of the target gene (5 pmol/*μ*L), 3 *μ*L of reverse primer (5 pmol/*μ*L), 4 *μ*L of cDNA TE buffer solution, and 15 *μ*L of RNase-free water were added. The denaturation conditions were 95°C for 15 s, the annealing conditions were 56°C for 30 s, and the elongation conditions were 72°C for 30 s. The fluorescence intensity of the amplification process was monitored using the My iQ single colour real-time RT-PCR detection system (Bio-Rad Laboratories). mRNA expressions were normalised using GAPDH.

### 2.8. Preparation of Membrane Fraction from Skin for Immunoblotting [15]

The skin tissue was homogenised in dissecting buffer (0.3 M sucrose, 25 mM imidazole, 1 mM EDTA, 8.5 *μ*M leupeptin, 1 *μ*M PMSF; pH 7.2) using a Physcotron homogeniser (Microtec Co., Ltd., Chiba, Japan) on ice. Each homogenate was homogenised using the ultrasonic homogeniser (UH-50, SMT Co., Ltd, Tokyo, Japan) and then centrifuged (4,000 × g for 15 min at 4°C). The resulting supernatant was centrifuged (200,000 × g for 60 min at 4°C). The supernatant was removed, and dissecting buffer was added to the precipitate. An additional homogenisation step was then performed using the ultrasonic homogeniser. The final homogenate contained the crude membrane fraction.

### 2.9. Electrophoresis and Immunoblotting

Protein concentrations were measured by the BCA method [16] using BSA as a standard. Electrophoresis was performed by Laemmli's method [17]. Using the loading buffer (84 mM Tris, 20% glycerol, 0.004% bromophenol blue, 4.6% SDS, and 10% 2-mercaptoethanol; pH 6.3), 6 *μ*g of protein was diluted 2-fold and applied to a polyacrylamide gel. After electrophoresis, the isolated proteins were transferred to a PVDF membrane. After blocking for 1 h using 1% skim milk, the resulting membrane was reacted for 1 h at room temperature with rabbit anti-rat AQP3 antibody (1/500). After washing the membrane with TBS-Tween (20 mM Tris-HCl, 137 mM NaCl, and 0.1% Tween 20; pH 7.6), the resulting membrane was reacted for 1 h at room temperature with anti-rabbit IgG-HRP antibody (1 : 2,000). After washing the membrane, the membrane was reacted with the ECL Plus detection reagent and then visualised with LAS-3000 mini (Fuji Film, Tokyo, Japan), a luminoimage analyser. 

### 2.10. Immunohistochemistry

Mice were anaesthetised with ether, and their hearts were perfused with PBS. They were then perfused with 50 mL of ice-cold 4% paraformaldehyde (PFA) in PBS. The skin was dissected out and post fixed in 4% PFA in PBS for 1 h at 4°C. Samples were immersed in 30% sucrose/PBS overnight at 4°C and embedded in OCT compound (Sakura Finetek USA Inc., Torrance, CA, USA). Frozen sections were cut with a cryostat (Leica Microsystems, Tokyo, Japan) at 10 *μ*m and mounted onto MAS-coated glass slides (Matsunami Glass, Osaka, Japan). Sections were washed with PBS and blocked with blocking solution (PBS containing 3% FBS and 0.1% Triton X-100) for 1 h; subsequently, sections were incubated overnight at 4°C in a mixture of rabbit anti-rat AQP3 antibody (1/200) or mouse anticeramide antibody (1/10). After washing 3 times with PBS, sections were reacted with secondary antibody (Alexa Fluor 488 anti-rabbit IgG, 1/200; Alexa Fluor 488 anti-mouse IgM antibody; 1/200) at room temperature for 1 h. After washing with PBS, sections were reacted with PI solution (1/1,000) in PBS at room temperature for 30 min, washed 3 more times with PBS, and then coverslipped with Vectashield (Vector Laboratories, Burlingame, CA, USA). Immunostained sections were observed under a microscope (BZ-9000, Keyence Corporation, Tokyo, Japan).

### 2.11. Statistical Analysis

Numerical data are expressed as the mean ± standard deviation. The significance of differences was evaluated using ANOVA, followed by Tukey's test. Values of *P* < 0.05 were considered significant.

## 3. Results

### 3.1. The Effect of Polyphenol-Rich Aqueous Extract of Acacia on Spontaneous Itching Behaviour

**Figure 1:**
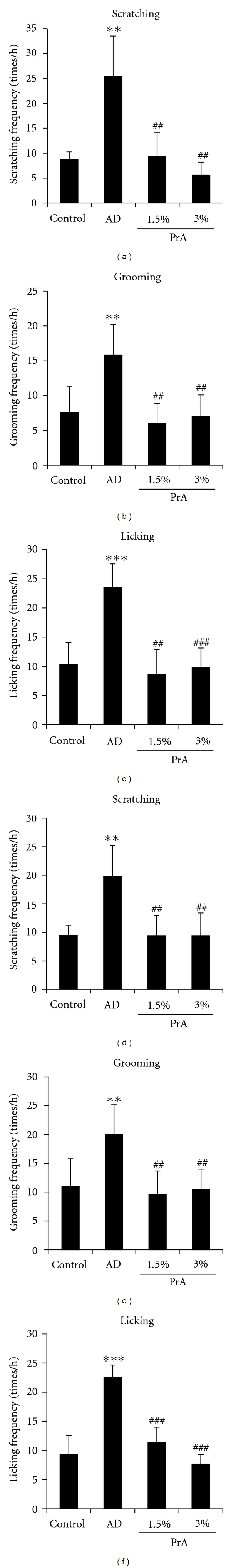
Analysis of spontaneous itching behaviour. HR-1 mice were given free access to a normal diet (control group), HR-AD diet (AD group), HR-AD diet with 1.5% PrA, or HR-AD diet with 3.0% PrA for 6 weeks. The spontaneous itching behaviour of mice was observed at 4 weeks (a)–(c) and 6 weeks (d)–(f) after the administration, and the frequency of spontaneous itching behaviour per hour was calculated. Data represent the mean ± SD for 8 mice. Tukey's test: **P < 0.01 and ***P < 0.001 versus control group, ^##^ *P* < 0.01 and ^###^ *P* < 0.001 versus AD group.

The frequency of spontaneous itching behaviour that occurred 4 and 6 weeks after the PrA administration is shown in Figure 1.

At 4 weeks after the administration, the frequency of scratching, grooming, and licking behaviour in the AD group was approximately twice that in the control group. The frequency of scratching, grooming, and licking in the PrA 1.5% and PrA 3.0% administration groups all decreased significantly compared to that in the AD group and was nearly equal to that in the control group (Figures 1(a)–1(c)). The same trends observed 4 weeks after the administration were also observed 6 weeks after the administration (Figures 1(d)–1(f)).

### 3.2. The Effect of Polyphenol-Rich Aqueous Extract of Acacia on the Dermal Water Content and the Transepidermal Water Loss

**Figure 2:**
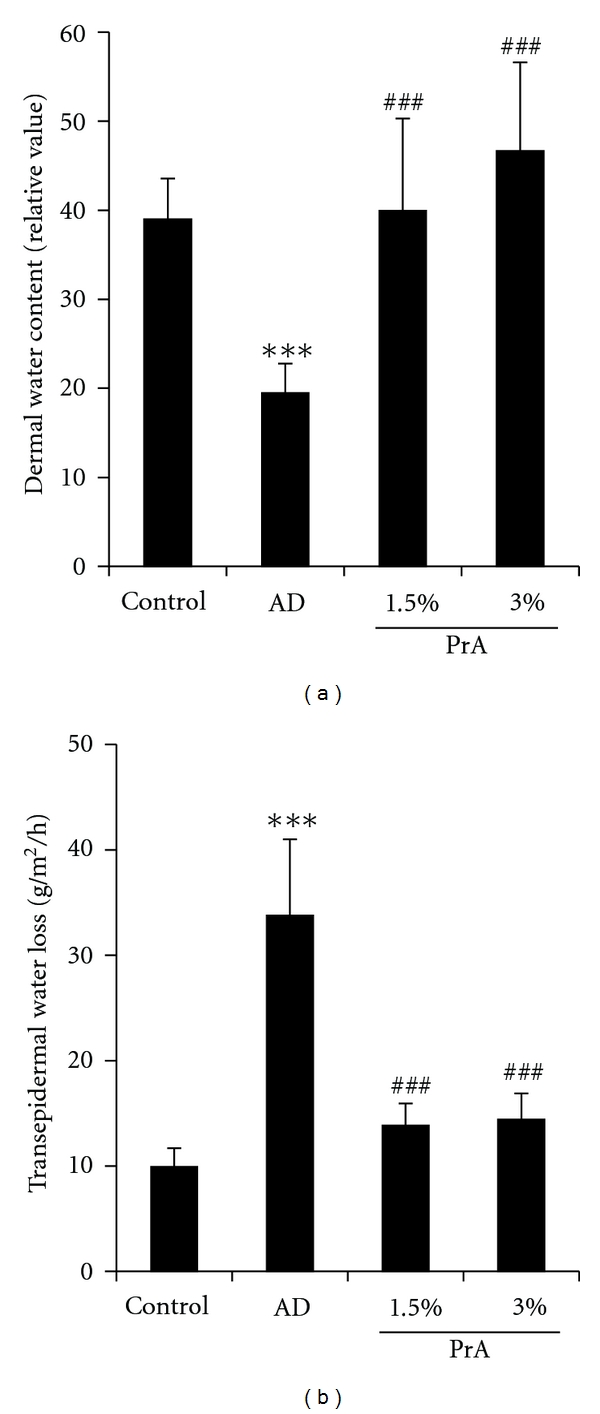
Dermal water content (a) and transepidermal water loss (b). HR-1 mice were given free access to a normal diet (control group), HR-AD diet (AD group), HR-AD diet with 1.5% PrA, or HR-AD diet with 3.0% PrA for 6 weeks. After 6 weeks, dermal water content (a) and transepidermal water loss (b) were measured. Data represent the mean ± SD for 8 mice. Tukey's test: ***P < 0.001 versus control group, ^###^ *P* < 0.001 versus AD group.

The dermal water content and the transepidermal water loss at 6 weeks after the administration are shown in Figure 2.

The dermal water content was significantly lower in the AD group than in the control group. The dermal water content was significantly higher in the PrA 1.5% and PrA 3.0% administration groups than in the AD group and was nearly equal to that in the control group (Figure 2(a)).

The transepidermal water loss in the AD group was approximately 3 times larger than that in the control group. The transepidermal water loss in the PrA 1.5% and PrA 3.0% administration groups was significantly smaller than that in the AD group and was nearly equal to that in the control group (Figure 2(b)).

### 3.3. The Effect of Polyphenol-Rich Aqueous Extract of Acacia on Aquaporin-3 (AQP3) Expression in the Skin

**Figure 3:**
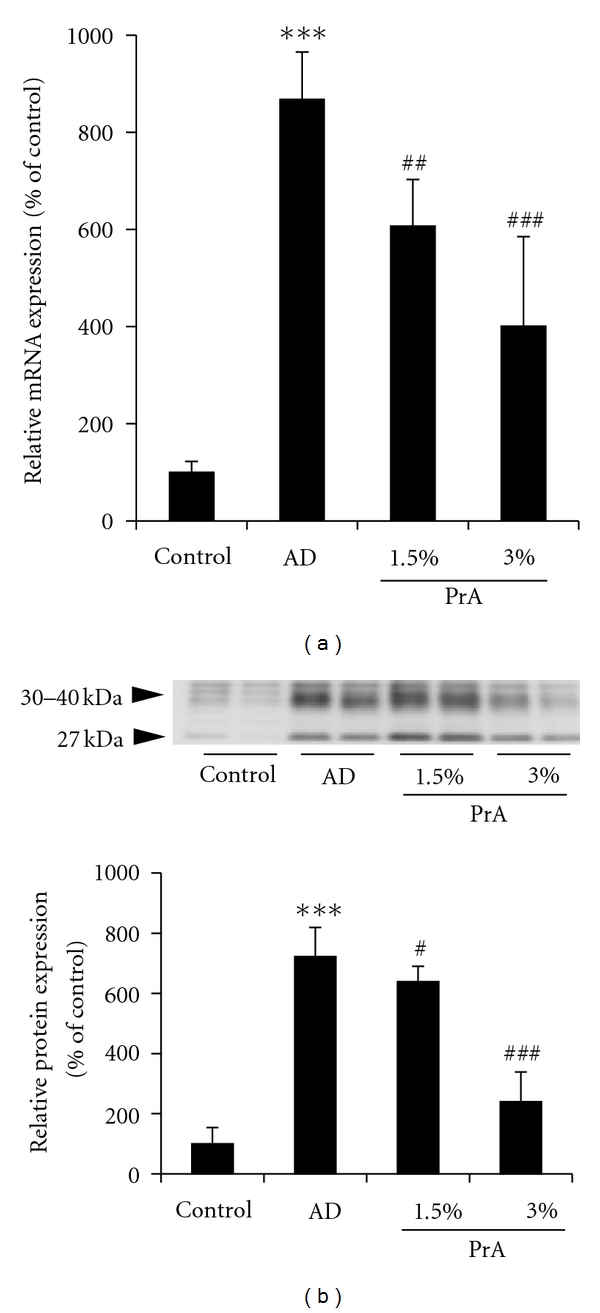
AQP3 mRNA (a) and protein (b) expression levels in the skin. HR-1 mice were given free access to a normal diet (control group), HR-AD diet (AD group), HR-AD diet with 1.5% PrA, or HR-AD diet with 3.0% PrA for 6 weeks. (a) The skin was harvested, and the AQP3 mRNA expression level was measured using real-time RT-PCR. GAPDH was used as a housekeeping gene. (b) The skin was harvested, and the AQP3 protein expression level was measured using Western blotting. mRNA and protein expression levels are presented as the percentage of the control group (100%). Data represent the mean ± SD for 8 mice. Tukey's test: ***P < 0.001 versus control group, ^#^ *P* < 0.05, ^##^ *P* < 0.01, and ^###^ *P* < 0.001 versus AD group.

The mRNA expression level and protein expression level of AQP3 in the skin are shown in Figure 3.

AQP3 expression increases in patients with atopic dermatitis [18, 19]. The mRNA expression level of AQP3 in the AD group was approximately 9 times larger than that in the control group. In the PrA 1.5% and PrA 3.0% administration groups, the mRNA expression level of AQP3 showed dose-dependent decreases (Figure 3(a)).

Two AQP3 protein bands were detected. One of these appeared at around 27 kDa and represented the deglycosylated form of AQP3, and the other appeared at around 30–40 kDa and represented a glycosylated form of AQP3 [20, 21]. The glycosylation is associated with the stability and intracellular translocation of AQP [22, 23], but it has no influence on water permeability [24]. In this study, therefore, the sum of these bands was analysed as the AQP3 protein expression level [25, 26]. The AQP3 protein expression level in the skin in the AD group was approximately 7 times larger than that in the control group. In the PrA 1.5% and PrA 3.0% administration groups, the AQP3 protein expression level was less than that in the AD group (Figure 3(b)).

**Figure 4:**
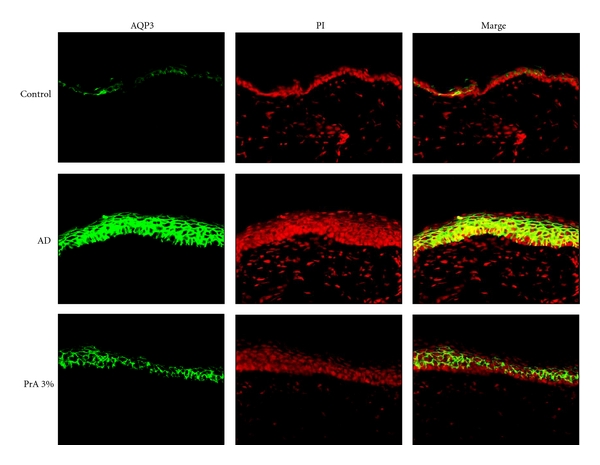
Analysis of the AQP3 expression distribution in the skin. HR-1 mice were given free access to a normal diet (control group), HR-AD diet (AD group), or HR-AD diet with 3.0% PrA for 6 weeks. After 6 weeks, the skin was harvested and AQP3 (green) and nuclei (red) were immunostained.

The AQP3 distribution was analysed by immunohistochemical staining (Figure 4). AQP3 was predominantly expressed in the stratum spinosum and stratum basale in the control group, in the AD group, and in the PrA 3.0% administration group. The AQP3 expression in the AD group was greater than that in the control group. The AQP3 protein expression level was clearly lower in the PrA 3.0% administration group than in the AD group.

### 3.4. The Effect of Polyphenol-Rich Aqueous Extract of Acacia on Ceramide Expression in the Skin

**Figure 5:**
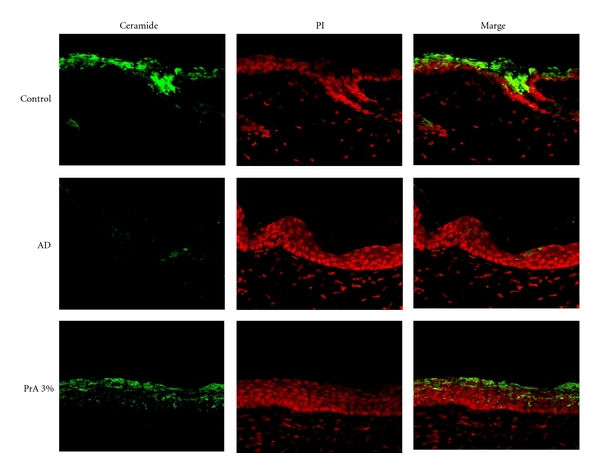
Analysis of the ceramide expression distribution in the skin. HR-1 mice were given free access to a normal diet (control group), HR-AD diet (AD group), or HR-AD diet with 3.0% PrA for 6 weeks. After 6 weeks, the skin was harvested and ceramide (green) and nuclei (red) were immunostained.

The ceramide distribution in the skin was analysed by immunohistochemical staining (Figure 5). In the control group, ceramide was widely distributed in the stratum corneum [27]. The ceramide expression level was clearly lower in the AD group than in the control group. The ceramide expression level in the PrA 3.0% administration group was nearly equal to that in the control group.

### 3.5. The Effect of Polyphenol-Rich Aqueous Extract of Acacia on the Expression of Enzymes That Increase or Decrease the Ceramide Content in the Stratum Corneum

**Figure 6:**
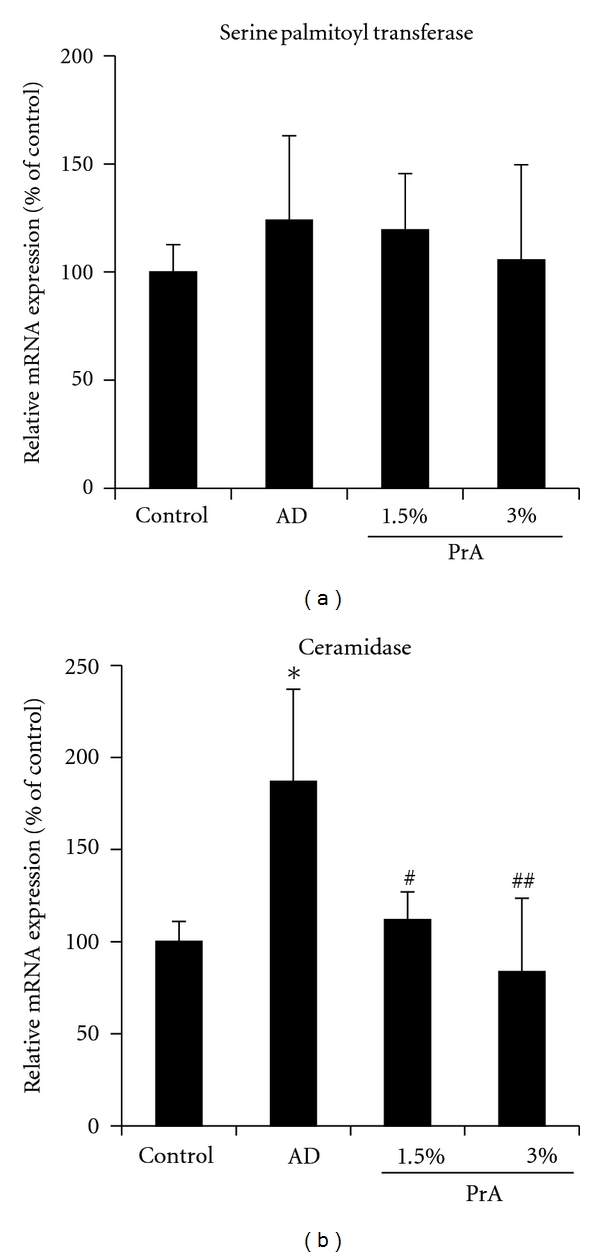
mRNA expression levels of serine palmitoyl transferase (a) and ceramidase (b) in the skin. HR-1 mice were given free access to a normal diet (control group), HR-AD diet (AD group), HR-AD diet with 1.5% PrA, or HR-AD diet with 3.0% PrA for 6 weeks. After 6 weeks, the skin was harvested, and serine palmitoyl transferase (a) and ceramidase (b) mRNA expression levels were measured using real-time RT-PCR. GAPDH was used as a housekeeping gene. mRNA expression levels are presented as the percentage of the control group (100%). Data represent the mean ± SD for 8 mice. Tukey's test: *P < 0.05 versus control group, ^#^ *P* < 0.05 and ^##^ *P* < 0.01 versus AD group.

The mRNA expression levels of serine palmitoyl transferase, which increases the ceramide content in the stratum corneum, are shown in Figure 6(a). No significant differences in the mRNA expression levels were observed among the control group, the AD group, and the PrA group (Figure 6(a)).

The mRNA expression levels of ceramidase, which decreases ceramide content in the stratum corneum, are shown in Figure 6(b). The mRNA expression level of ceramidase in the AD group was approximately twice that in the control group. This increase was successfully inhibited by PrA administration (Figure 6(b)).

## 4. Discussion

Atopic dermatitis is a skin disease that is characterised by a course marked by exacerbations and remissions of pruritic eczema. The pruritus of atopic dermatitis is a refractory symptom that generally cannot be controlled with antihistamines. In this study, a mouse model of atopic dermatitis with pruritic symptoms was prepared by giving mice a special diet (Table 1) that contained no magnesium according to the method reported by Fujii et al. [12, 13]. Spontaneous itching behaviour was evaluated by observing scratching, grooming, and licking behaviour. An increase in the spontaneous itching behaviour was observed in the AD group compared to the control group; thus, it was confirmed that the itching associated with atopic dermatitis could be properly evaluated in this animal study (Figure 1). In the PrA administration groups, a decrease in the spontaneous itching behaviour was observed (Figure 1). These results confirm the inhibitory effect of PrA on itching associated with atopic dermatitis.

Non allergic factors such as skin dryness are involved in the development of pruritus associated with atopic dermatitis. In the AD group, a decrease in the dermal water content and an increase in the transepidermal water loss were observed (Figure 2). Therefore, it was suggested that the pruritus observed in this animal study was caused by skin dryness. It is considered that PrA may be regulating the dermal water content because PrA exhibited an inhibitory effect on the above-mentioned skin dryness (Figure 2).

AQP3 [28] and ceramide [29, 30] play important roles in regulating the dermal water content. A decrease in the AQP3 expression level in normal skin causes skin dryness, as AQP3-knockout mice have reduced dermal water content [28]. In addition, reduced stratum corneum ceramide content causes an increase in transepidermal water loss, leading to reduced dermal water content [29, 30]. Therefore, it is considered that a normal state of ceramide and the existence of a normal or higher AQP3 level are the essential conditions for maintaining the normal water content of the skin. In atopic dermatitis, tumour necrosis factor-*α*, which is secreted by keratinocytes, increases the production of chemokine CCL17, causing an increase in the AQP3 expression level [18, 31]. However, it is also known that a decrease in the ceramide content in the stratum corneum associated with atopic dermatitis causes an increase in transepidermal water loss, leading to a decrease in dermal water content [29, 30]. In the AD group, the AQP3 expression level was higher than that in the control group (Figures 3 and 4) and the ceramide expression was lower than that in the control group (Figure 5). It is considered that, since the increase in AQP3 expression level in the AD group takes place under the condition of a decreased stratum corneum ceramide content, transepidermal water loss increased, leading to a decreased stratum corneum water content and an increase in spontaneous itching behaviour. In the PrA group, it is considered that transepidermal water loss and the associated spontaneous itching behaviour were reduced due to a normal level of stratum corneum ceramide content, although the AQP3 expression level was slightly increased compared to that in the control group (Figures 3, 4, and 5).

In the skin, ceramide is synthesised from palmitoyl CoA and by serine palmitoyl transferase in the cells of the stratum basale [32]. The synthesised ceramides move toward the stratum corneum with skin cell differentiation and construct the lamellar structure of the stratum corneum. At the same time, ceramides in the stratum corneum are degraded by ceramidase into sphingosine and fatty acids [33]. Therefore, the stratum corneum ceramide content reflects the balance between the ceramide synthesis by serine palmitoyl transferase and its degradation by ceramidase [34]. In atopic dermatitis model mice, the ceramide content is decreased due to the increased expression of ceramidase in the stratum corneum [27]. In the AD group in this study, the expression level of ceramidase was increased and the ceramide content in the stratum corneum was reduced (Figures 4 and 6). The stratum corneum ceramide content in the PrA group was nearly equal to that in the control group, which may be because PrA inhibited an increase in the expression of ceramidase (Figures 4 and 6).

It has been reported that catechins such as tea polyphenol [9, 10] and apple polyphenol [11] are effective for treating atopic dermatitis to some extent. It has been revealed that the mechanism of this effect includes the inhibition of IgE production [35], the inhibition of the release of inflammatory substances such as histamine from mast cells [36, 37], and the inhibition of the expression of high affinity IgE receptor Fc*ε*RI in mast cells [38]. Because PrA is also a polyphenol, it is possible that PrA inhibits the development of atopic dermatitis by these mechanisms. It is considered necessary to examine in detail whether PrA inhibits the development of allergic dermatitis.

In summary, it has been revealed that PrA improves the itching associated with atopic dermatitis by preventing the skin from drying. It has also been revealed that PrA prevents the skin from drying by inhibiting the decrease in ceramide content in skin affected by atopic dermatitis. Moreover, it has been revealed that PrA maintains the ceramide content in the skin because PrA inhibits the increase in ceramidase expression caused by atopic dermatitis. In the future, PrA is expected to offer potential as a plant extract that is useful for alleviating atopic dermatitis.

##  Conflict of Interests

The authors have declared that there is no conflict of interests.

## Figures and Tables

**Table 1:** Ingredients of normal and HR-AD diets for HR-1 hairless mice.

	Normal diet	Special diet
General ingredients		
Moisture	9.2%	1.6%
Crude protein	18.8%	21.6%
Crude fat	3.9%	0.1%
Crude fibre	6.6%	4.4%
Crude ash	6.9%	5.6%

Minerals		
Ca	1.0%	0.9%
P	0.8%	0.8%
Mg	0.3%	0.0%
K	1.0%	0.4%
Na	0.3%	0.2%
Fe	240 mg/kg	277 mg/kg
Mn	101 mg/kg	80 mg/kg
Zn	89 mg/kg	123 mg/kg
I	0.77 mg/kg	0.22 mg/kg
Co	0.08 mg/kg	0.002 mg/kg
Cu	14 mg/kg	22 mg/kg

Vitamins (1 kg)		
A	16150 IU	32157 IU
D_3_	3169 IU	4799 IU
E	45 mg	160 mg
K	15 mg	5 mg
B1	11 mg	13 mg
B2	9 mg	16 mg
B6	19 mg	53 mg
B12	0.03 mg	0.08 mg
Biotin	0.3 mg	0.8 mg
Pantothenic acid	25 mg	30 mg
Choline	1820 mg	868 mg
Folic acid	2.6 mg	0.1 mg
Inositol	10 mg	unknown
Niacin	102 mg	320 mg

**Table 2:** Primer sequences of mouse mRNA.

Target	Accession number	Forward primer (5′ to 3′)	Reverse primer (5′ to 3′)
AQP3	NM_016689	CTCATGTTGTGCCAGAGTGTG	CCATCAGCAAGCCTAGAAGTC
serine palmitoyl transferase	NM_011479	CTGTGCTCACATATGTGGGCT	CTTTCTGTTGCATGGTGGCAC
ceramidase	NM_019734.2	GCAGAACACCGGCCAAG	CAGTCAGCTTGTTGAGGACAG
GAPDH	NM_008084	GGCAAATTCAACGGCACAGT	AGATGGTGATGGGCTTCCC
